# Long-Term Determinants of Depression Mood: A 19-Year Follow Up of 2344 Middle-Aged and Older Adults

**DOI:** 10.3390/healthcare12242568

**Published:** 2024-12-20

**Authors:** Nae-Fang Miao, Chia-Jung Hsieh, Pei-Shan Li

**Affiliations:** 1 Post-Baccalaureate Program in Nursing, College of Nursing, Taipei Medical University, Taipei 110301, Taiwan; naefang@tmu.edu.tw; 2 School of Nursing, College of Nursing, National Taipei University of Nursing and Health Sciences, Taipei 112303, Taiwan; 3 Department of Long-Term Care, College of Health Technology, National Taipei University of Nursing and Health Sciences, Taipei 112303, Taiwan; michelle19800130@gmail.com

**Keywords:** older adults, depression, protective factors, risk factors, cognitive function

## Abstract

**Objectives:** This study explores depression changes and influencing factors in middle-aged and older individuals, focusing on different cognitive function states. **Methods:** This cohort study uses panel data from seven waves of the nationally representative Taiwan Longitudinal Study on Aging (1996–2015) with 2344 participants aged 50 or above. Data analysis was conducted from 25 January 2023 to 4 May 2023. **Results:** Multivariate logistic regression analyzed three trajectories: maintained mood, progressive depression, and consistent depression. Protective factors for progressive depression included self-health perception, exercise, family satisfaction, and financial well being, while risk factors were chronic diseases, pain, substance use, and daily activity limitations. Cognitive function emerged as a significant protective factor, while pain exhibited the highest risk. In the cognitive dysfunction group, only social participation showed notable differences. **Conclusion:** Progressive and consistent depression in middle-aged and older people have aroused concern. In addition to necessary protection and risk factors, special attention should be paid to the risks posed by the level of pain. Addressing pain as a crucial risk factor is essential, particularly for middle-aged and older people with cognitive impairment and depression, necessitating targeted support.

## 1. Introduction

The World Health Organization, in its global health estimates, emphasized that depression’s prevalence varies across age groups, with a peak among middle-aged and older populations [1]. Depression manifestations and risk factors demonstrate notable variations across different life stages, While younger adults tend to present with higher rates of comorbid drug abuse, personality disorders, and cognitive symptoms, middle-aged individuals more commonly experience recurrent major depressive disorder characterized by physical comorbidities and neurovegetative symptoms. [2], and elderly populations often present with more complex problems. Other previous findings reveal that older adults with depression tend to exhibit higher levels of anhedonia than younger adults, with elderly populations potentially experiencing higher rates of somatic symptoms, social isolation, and comorbid medical conditions that can mask or complicate traditional diagnostic criteria [3]. This has made depression a primary mental health challenge, notably affecting cognitive function in older adults [4]. Consequently, a public health perspective has drawn attention to the increasing rates of depression among older adults and the complexities associated with effectively managing it [5]. According to the Ecological Systems Theory, an individual’s development is influenced by all aspects of their environment [6]. There are case studies applying this theory to analyze and implement various systems for older adults dealing with depression [7]. Hence, it can be inferred that the personal, familial, and social–environmental facets of Ecological Systems Theory play a pivotal and influential role in the experience of geriatric depression.

Current research highlights risk factors for geriatric depression, including advanced age, female gender, lower education, economic hardship, sedentary lifestyle, smoking, alcohol consumption, multiple illnesses, perceived deteriorating health, cognitive function, insomnia, worsening instrumental activities of daily living (IADL), and strained family interactions. Protective factors encompass higher educational attainment, engagement in physical activity, participation in skill-enhancing courses, computer usage, family and social support, and involvement in social activities [8,9,10,11]. However, not all commonly cited factors can universally increase or mitigate the risk of depression in older populations. Furthermore, a consensus on specific factors remains elusive [9]. Although the existing literature may not provide a definitive grasp of the extent to which these protective and risk factors impact geriatric depression, it is crucial to consider testing them against the backdrop of the Ecological Systems Theory.

The process of aging detrimentally impacts cognitive function, with memory and reasoning experiencing accelerated decline, which is particularly notable [12]. Additionally, social isolation and loneliness in old age pose a distinct risk for cognitive impairment, which serves as a crucial marker for cognitive decline [13]. Interestingly, the protective factors and risk factors associated with depression in old age appear to intersect with those of cognitive impairment. Cognitive impairment emerges as a central feature of the clinical manifestations of depression [14]. While previous research has acknowledged the association between cognitive function decline due to aging and depression as a relevant risk factor for cognitive deterioration [4], the extent to which cognitive function is linked to varying degrees of depression in the older population fluctuates over time. Thus, an urgent research priority is to ascertain the protective and risk factors influencing the degree to which cognitive function is impacted during different stages of geriatric depression.

Hence, the purpose of this research was to undertake a 19-year prospective investigation utilizing data from the “Taiwan Longitudinal Study on Aging (TLSA)” conducted by the Taiwan Ministry of Health and Welfare. The research aims to fill existing knowledge gaps regarding geriatric depression by conducting a comprehensive longitudinal study to identify protective and risk factors influencing the progression of depression and explore their impact on the trajectory of depression development in middle-aged and older individuals with varying cognitive function statuses.

## 2. Method

### 2.1. Materials and Methods

Data sources. This was a retrospective population-based fixed-cohort study that employed the Taiwan Longitudinal Study on Aging (TLSA) as its primary data source. This survey database on the health and living conditions of older adults is a government-level database. It has been planned since 1987 and is conducted every four years. The survey was conducted using a wide range of follow-up interviews, and the relevant measurement tools were all self-report questionnaires. All included questionnaire types were discussed, added, deleted, and revised through focus groups by a team of experts and scholars in the related fields of geriatrics, psychometrics, and sociology invited by the government. The entire questionnaire had good expert face validity and content validity. However, for the older-age group, the questionnaire design has a shorter interview time and stress load when answering questions, but it also needs to consider the factors of cognitive decline. For such a purpose, self-assessed health status, exercise behavior, family living satisfaction, social participation situation, economic satisfaction, the number of chronic diseases diagnosed by doctors, and oral pain experience are all suitable.

Participants. This study utilized data from the Longitudinal Survey of Health and Living Status of the Elderly in Taiwan, which began in 1989 as a nationally representative sample of individuals aged 60 and older. The research employed a multi-stage sampling approach, including all individuals aged 60 and over in Taiwan’s non-aboriginal areas, as per the household registration system at the close of 1996. The same sampling procedure was followed for the 1996 survey. Individuals who did not complete the survey that year were excluded from this study. Ultimately, a cohort of 2344 subjects aged 50 to 66 years was included in the analysis for this research.

### 2.2. Procedures

This longitudinal study was conducted over six waves spanning 19 years, with the final analysis conducted using data from 2015 (see Supplementary Figure S1). A succinct overview of this research is provided here, with comprehensive details available elsewhere [15]. The participants of this study were drawn from the Longitudinal Survey of Health and Living Status of the Elderly in Taiwan, which initially surveyed individuals aged 60 years and older. The sampling methodology used a multi-stage approach, covering non-aboriginal areas of Taiwan based on the household registration system as of 1996. For the current analysis, we focused on data from 2344 participants aged 50–66 who were surveyed in 1996 and followed through to 2015. The outcome of the present study was the presence of depressive symptoms measured with the 10-item version of the Center for Epidemiologic Studies-Depression (CES-D 10) scale [16]. Each item on the CES-D is coded for the number of days the participant experienced depressive symptoms during the last week: “0” for 0 days, “1” for 1 day, “2” for 2–3 days, and “3” for 4 days or more. The total score range is 0–30, with a higher score indicating more depressive symptoms. The cutoff score of 10 on the TLSA form of the CES-D is, therefore, recommended for screening for depressive symptoms in older adults in Taiwan, as the scale has good sensitivity and specificity for older Chinese individuals [17]. Cronbach’s alpha for the CES-D score in this study was 0.93, demonstrating good internal consistency.

Six protective factors were considered in this study. Self-rated health (SRH) was measured by a single item, “Regarding your state of health, do you feel it is very poor, not so good, average, good, or excellent?” Responses were scored from 1 to 5, with higher scores indicating a better or more positive SRH [18]. The routine exercise behavior survey item assessed participants’ exercise habits with the question “Do you usually exercise?” Response options included “No”, “2 times/week”, “3–5 times/week”, and “6 times/week”. A higher score indicated a stronger inclination towards regular exercise. Family living satisfaction assesses the level of satisfaction of research subjects with their current living arrangements with family members. A score of 1 indicates a high level of dissatisfaction, while a score of 5 indicates satisfaction. A higher score reflects greater satisfaction with living arrangements with family members. Cognitive function was assessed using the Chinese SPMSQ scale. Cognitive status was classified into two groups: “normal mental functioning” (SPMSQ score 8 and higher) and “cognitive impairment” (score 7 and lower), which includes mild, moderate, and severe cognitive impairment [19]. This assessment of social participation primarily inquires whether the research subjects have engaged in activities organized by social groups (such as social service or public welfare groups, older adult learning groups, or activities within senior clubs) in the past six months. It also calculates the frequency of their participation in group activities. A higher score indicates a higher level of social participation. The financial satisfaction question aims to evaluate individuals’ satisfaction with their current economic circumstances, utilizing a 5-point self-evaluation scale. A rating of 1 represents “very dissatisfied”, 2 corresponds to “dissatisfied”, 3 signifies “average”, and 4 indicates “satisfied”. A rating of 5 reflects “very satisfied”.

Four risk factors were examined in this study. A higher count of chronic diseases diagnosed by a doctor indicates a greater burden of chronic illnesses on the individual. Physical health status was assessed through self-reported pain and bodily sensations experienced during the month prior to assessment. A score of 0 signifies no pain, a score of 1 represents slight pain, and a score of 2 indicates moderate pain. A score of 3 corresponds to severe pain, with higher scores indicating a more intense pain experience for the individual [20]. Substance use was assessed using three items: smoking, alcohol consumption, and betel nut chewing, and each categorized as “yes” or “no” based on the subject’s self-report of their substance use habits in the previous 6 months. These responses were then scored from 3 to 0, with higher scores indicating a greater likelihood of using addictive substances. Instrumental Activities of Daily Living (IADL) limitations were assessed by asking whether they had difficulty with six tasks involving social interaction (shopping, using the telephone, handling finances, performing heavy or light housework, and transportation). Higher IADL scores indicated more limitations in daily activity [21].

### 2.3. Statistical Analysis

The statistical progressions of study variables are described for both the study baseline and follow-up periods. The data analysis was conducted in SPSS (v.19, IBM Corp., Armonk, NY, USA), with a predetermined statistical significance level of α = 0.05. Descriptive statistics are provided as the mean and standard deviation (SD) for continuous variables and as frequency and proportions for categorical variables. To assess the impact factor’s strength, Multinomial Logistic Regression was employed for scenarios involving more than two types of dependent variables. The odds ratio (OR) was utilized for this evaluation. If the 95% confidence interval (CI) of the odds ratio did not encompass 1, it indicated statistical significance (*p* < 0.05). Trajectory model analysis is conducted using the SAS procedure “PROC TRAJ” (version 9.4) (SAS Institute Inc., Cary, NC, USA) developed by Jones and Nagin [22].

## 3. Result

### 3.1. Sample Characteristics

The respondent indicators of this longitudinal survey are listed in Table 1. In all, 2344 subjects were analyzed to identify the effects of the factors of the depression process on three depression trajectories in Taiwan. One particularly notable feature was the extraordinarily high response rate that was achieved from the baseline study to follow ups.

### 3.2. Depression Trajectories

Table 2 presents the analysis of 2344 subjects who were examined for model testing regarding depression trajectories across two to five classes among a sample of older individuals in Taiwan who were followed for over 19 years (1996–2015). After evaluating the indices for determining the optimal number of depression trajectories (DTs), we observed that the model with three depression trajectories exhibited the best fit, as indicated by the significant decrease in Bayesian Information Criteria (BIC) and Akaike Information Criterion (AIC) values.

**Figure 1:**
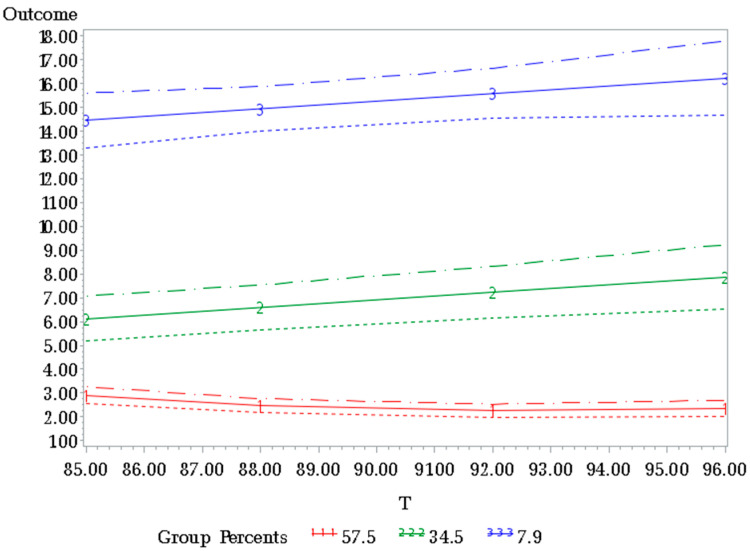
Depression trajectories among a sample of older individuals that survived a 19-year period in Taiwan. The X-axis is the survey time (years) and the Y-axis is the depression level. The solid line indicates the sample trajectories and the upper and lower dashed lines indicate the 95% confidence limits of the estimated trajectory, representing the statistical uncertainty in the estimation. The membership probabilities of the three depression trajectories are as follows: maintained mood (57.5%); progressive depression (34.5%); and consistent depression (7.9%).

Figure 1 illustrates the membership probabilities and the patterns of depression trajectories (DTs). These trajectories encompass three distinct subtypes as follows: trajectory 1—maintained mood (57.5%); trajectory 2—progressive depression (34.5%); and trajectory 3—consistent depression (7.9%). The subgroup within the maintained mood trajectory exhibited consistently low depression levels throughout the 19-year period. In contrast, the subgroup within the progressive depression trajectory initially displayed relatively low depression levels, which then escalated rapidly as the study period progressed. Finally, the subgroup within the consistent depression trajectory steadily maintained a higher level of depression, which remained relatively constant until the end of the observation period.

### 3.3. Protective and Risk Factors

Table 3 presents the results of time-constant variables and time-varying variables (including protective and risk factors) in predicting different trajectories among older adults. For trajectory 1 (maintained mood), trajectory 2 (progressive depression), and trajectory 3 (consistent depression), statistically significant differences were observed only in protective factors, including self-health perception (F = 193.88, *p* < 0.01), family living satisfaction (F = 72.32, *p* < 0.01), cognitive function (F = 28.25, *p* < 0.01), and social participation (F = 18.40, *p* < 0.01). No significant differences were found among the risk factors.

### 3.4. Predictive Factor for Escalating Depression Trajectory

As shown in Supplementary Table S1, with adjustments for demographic variables, including gender, education level, and living alone, the comparison between trajectory 2 (progressive depression) and trajectory 1 (maintained mood) revealed ORs for both protective and risk factors. Specifically, among the protective factors, self-health perception (OR = 0.74, *p* < 0.001), exercise behavior (OR = 0.75, *p* < 0.01), family living satisfaction (OR = 0.76, *p* < 0.001), and financial satisfaction (OR = 0.74, *p* < 0.001) were associated with a decreased risk of progressive depression for each one-unit increase. Additionally, among the risk factors, an increase in the number of chronic diseases (OR = 1.19, *p* < 0.001), pain (OR = 1.35, *p* < 0.001), substance use (OR = 1.16, *p* < 0.05), and IADL difficulty (OR = 1.08, *p* < 0.05) was linked to an increased risk of progressive depression for each one-unit increase. Notably, among these factors, pain had the highest OR.

After adjustments for demographic variables, such as gender, education level, and living alone, the comparison between trajectory 3 (consistent depression) and trajectory 1 (maintained mood) revealed ORs for both protective and risk factors. Specifically, among the protective factors, self-health perception (OR = 0.53, *p* < 0.001), exercise behavior (OR = 0.62, *p* < 0.05), family living satisfaction (OR = 0.46, *p* < 0.001), cognitive function (OR = 0.87, *p* < 0.05), and financial satisfaction (OR = 0.38, *p* < 0.001) were associated with a decreased risk of consistent depression for each one-unit increase. Additionally, among the risk factors, an increase in the number of chronic diseases (OR = 1.41, *p* < 0.001), pain (OR = 1.95, *p* < 0.001), or IADL difficulty (OR = 1.14, *p* < 0.05) was linked to an increased risk of consistent depression for each one-unit increase. Notably, among these factors, the OR of pain was also the highest. It is worth noting that a new factor, cognitive function, was added to the protective factors, as this analysis indicated that it played a significant protective role.

### 3.5. Factors Varying Depression Trajectories in Different Cognitive States

The results of both time-constant variables and time-varying variables (including protective and risk factors) in predicting different trajectories among older adults are displayed in Table 4. Among subjects within the cognitively normal group, significant differences were observed across the three distinct depressive trajectories. Specifically, among the protective factors, there were notable differences in self-health perception, exercise behavior, family living satisfaction, social participation, and financial satisfaction. In terms of risk factors, there were significant differences in the number of chronic diseases, pain, and IADL difficulty.

However, among the three distinct depressive trajectories within the cognitive impairment group, significant differences were observed in various protective and risk factors. Among the protective factors, self-health perception, exercise behavior, family living satisfaction, and financial satisfaction displayed significant differences. Likewise, among the risk factors, significant differences were noted in a number of chronic diseases, pain, and IADL difficulty. Notably, in the original protective factor variables, the factor of social participation, which plays a pivotal role in the degree of cognitive function’s influence on depressive trajectories, exhibited non-significance.

## 4. Discussion

The depressive mood trajectories identified in our research exhibit similarities to those observed in older adults from various countries, typically categorized into three or four distinct trajectory groups [23]. However, our research encompassed a longer observational period compared to other investigations. Furthermore, we observed a consistent pattern in these studies that examine depression trajectory trends in older age. The majority of participants exhibited either no depressive symptoms or maintained emotional stability. Conversely, a subset of participants experienced a progression towards increasingly severe depressive symptoms. These shared findings, both from our research and other cohort studies, are in line with the conclusions drawn from systematic reviews [23]. However, none of the trajectory patterns indicated a shift from depressive symptoms to a non-depressive state over time. Our research reveals the presence of persistent and escalating depressive symptoms during the aging process, underscoring the need for heightened attention to the mental health and emotional well being of older individuals.

Our findings indicate that the factors influencing depression among middle-aged and older adults are distinct from those in other populations and follow diverse trajectories. While previous longitudinal research explored the influence of sporadic health-related factors, the existing literature has offered limited insights into the impact of protective factors, like family life satisfaction and financial well being, on depression trajectories. Particularly noteworthy is the concept of filial piety within ethnic Chinese culture, which stipulates that adult children must respect and care for parents unable to support themselves [24]. This filial responsibility is not only deeply ingrained in ethnic Chinese culture but also mandated by law in various Asian countries as a means of supporting and caring for older parents [25]. Consequently, this cultural context offers a degree of financial protection to ethnic Chinese older adults, making family-related factors a fundamental source of support [26].

However, this unique cultural element also contributes to the distinct vulnerability of ethnic Chinese individuals to geriatric depression. Filial piety promotes intergenerational solidarity and ensures care for older parents, which can lead to financial stability and emotional security for those unable to support themselves [27]. However, its rigid or authoritarian interpretation can create high expectations for children, particularly in families with limited resources. These expectations may exacerbate feelings of stress, guilt, or inadequacy when children struggle to meet familial obligations. For parents, reliance on filial piety may create vulnerability if their expectations for care are unmet, potentially leading to feelings of disappointment or depression [28]. Thus, the interaction between cultural norms, family dynamics, and available resources determines whether filial piety acts as a protective factor or contributes to psychological vulnerabilities. Additionally, in older adults, living with their families has been associated with lower depression morbidity rates. Our research confirms the specific protective role of family-related factors under the influence of ethnic Chinese culture.

Identifying factors that may influence the trajectory of mood frailty is essential, framing the interplay between individual change and the environment through the lens of the Ecological Systems Theory [6]. Our 19-year study on middle-aged and older individuals’ depression differs from other studies, especially in considering additional protective and risk factors. As individuals age, the perception of declining motor skills, increased prevalence of chronic diseases, heightened frequency of pain, and difficulties in instrumental activities of daily living (IADL) tend to intensify. These factors, in turn, exhibit a robust association with geriatric depression. Aging also contributes to social isolation, loneliness, illness, and negative emotions, which in turn escalate the use of tobacco and alcohol. These protective and risk factors for middle-aged and older individuals could underpin depression [29]. This finding aligns with previous systematic reviews that have documented similar patterns [9]. Longitudinal trajectory studies approach research differently than cross-sectional studies, focusing more on the evolution of subjects’ outcomes over time, rendering them more dynamic.

Considering the 19-year duration of this study’s follow up, and after controlling for demographic variables, it is evident that certain protective and risk factors play roles in influencing depression among middle-aged and older individuals. Our study conducted a comprehensive examination of these overlooked aspects. Within the framework of ethnic Chinese culture’s traditional norms, it is customary for children to cohabit with their parents and fulfill both material and non-material care responsibilities, impacting the overall contentment of middle-aged and older ethnic Chinese individuals with their family living arrangements and financial circumstances [30]. From a neurobiological perspective, both physical pain and the emotional distress of depression trigger similar brain regions [31]. This could potentially explain the high comorbidity rate between pain and depression in middle-aged and older populations. This research has also highlighted the significant protective role played by cognitive function in persistent depression trajectories. Depressive symptoms in late life may serve as an early indicator of cognitive decline, often accompanied by common brain lesions and elevated vascular risk factors [32]. Hence, it is crucial to assess cognitive impairments and abnormal brain changes in middle-aged and older individuals to detect depression early and provide timely intervention and treatment.

Specifically, cognitive function emerged as a newfound protective factor within the consistent depression trajectory model. Remarkably, when examining middle-aged and older individuals with both normal and abnormal cognitive functions, nearly all protective and risk factors exhibited a significant influence on the three different depression trajectory patterns. Intriguingly, among the protective factors, the role of social participation wielded a significant effect only in cases where cognitive function was normal. Systematic reviews have also indicated an inconsistent association between social networks and dementia [33]. From a pathological perspective, neuroimaging studies have unveiled distinct brain alterations in individuals experiencing depression [34]. Furthermore, cognitive decline is often intertwined with vulnerable areas associated with memory. Lesions in these areas perturb the neural networks underpinning cognition, memory, and behavior [35]. This cascade of evidence solidifies depression’s role as a potential causal factor in subsequent cognitive decline. Hence, we can surmise that during the confluence of depression and abnormal cognitive function, individuals’ capacity to actively engage in social interactions and community participation is impaired. It is crucial to assess cognitive impairments and abnormal brain changes for the early detection of depression and timely intervention. For middle-aged and older individuals dealing with both cognitive impairment and depression, a multifaceted approach is crucial. This includes health education from midlife to encourage physical activity, ensure financial stability, and manage chronic illnesses and pain conditions.

### Advantages and Limitations

This research boasts several notable strengths. Foremost, underpinned by the Ecological Systems Theory framework, we delve into the significance of pertinent factors that impact depression in middle-aged and older adults, considering factors at an ecological level. Notably, we shed light on aspects that have been historically overlooked, such as household family satisfaction, financial contentment, and pain levels. Moreover, our study employs innovative analytical methodologies to explore intricate depression trajectories across varying cognitive states, adding depth to findings as we examine the interplay between protective and risk factors. We also acknowledge several limitations in this study. First, we lack data to determine whether participants were diagnosed with depression and received appropriate medication during the follow-up period. Secondly, our sample mainly consisted of relatively healthy community-dwelling middle-aged and older individuals, potentially limiting the generalizability of our findings to those in residential institutions or long-term care settings. Furthermore, the definition of “social participation” was constrained by the database, focusing primarily on activities designed for seniors. Future research should address this limitation by investigating participation in intergenerational activities—such as sports, educational programs, community service, advocacy, or travel—which could foster inclusive social networks, enhance emotional well being, and reduce depression through intergenerational engagement. Despite these limitations, our utilization of a representative sample in a long-term longitudinal follow-up study to uncover the protective and risk factors influencing depression trajectories contributes valuable insights.

## 5. Conclusions

This study significantly advances our comprehension of the trajectory of depression development in middle-aged and older individuals. It holds vital implications for the design of intervention and support programs tailored for the middle-aged and older demographic. By incorporating these insights, we can enhance mental well being while concurrently slowing the pace of cognitive decline. Future research and clinical endeavors should delve deeper into the integration of protective factors, the mitigation of risk factor effects, and the strategic introduction of interventions at opportune junctures. This approach lays the foundation for a healthier and more content later life for middle-aged and older individuals.

## Figures and Tables

**Table 1:** Baseline sample characteristics (*N* = 2344).

Variables	*n*	%	Mean	SD
Age (at 1996)				
Middle age	2168	92.5		
Old age	176	7.5		
Gender				
Male	1205	51.4		
Female	1139	48.6		
Education				
Illiteracy	584	24.9		
Literacy	1760	75.1		
Living alone				
Non	2212	94.4		
Yes	131	5.6		
Missing	1	0		
Protect factors				
Self-health perception			3.34	1.11
Exercise behavior			0.46	0.50
Family living satisfaction			4.06	0.76
Cognitive function _Recall			4.67	2.26
Social participation			0.60	0.88
Financial satisfaction			3.18	0.87
Risk factors				
Number of chronic diseases			1.01	1.22
Pain			1.47	0.75
Substance use behavior			0.64	0.88
IADL difficulty			0.56	1.94
Depression				
Depression_1996			5.01	5.48
Depression_1999			4.51	5.41
Depression_2003			4.85	5.49
Depression_2007			4.93	5.78
Depression_2011			5.09	6.00
Depression_2015			5.29	5.76

**Table 2:** Depression trajectories.

No. of Trajectories	BIC	AIC	L	Membership Probabilities in the Models				
2	−28,213.28	28,193.02	28,186.02	1851 (75.18)	611 (24.82)			
3	−21,054.18	21,025.24	21,015.24	1516 (61.58)	764 (31.03)	182 (7.39)		
4	−28,004.27	27,946.39	27,926.39	330 (13.4)	503 (20.43)	1484 (60.28)	145 (5.89)	
5	−27,967.88	27,921.57	27,905.57	424 (17.22)	179 (7.27)	1479 (60.07)	261 (10.6)	119 (4.83)

Note. Model testing for depression trajectories across two to five classes among a sample of older individuals who survived over 19 years in Taiwan. (1996~2015); AIC = Akaike Information Criterion.; BIC = Bayesian Information Criteria. L: Log-Likelihood.

**Table 3:** Protect and risk factors by three trajectory groups of depression.

	Trajectory of Maintained Mood (*n* = 1516)		Trajectory of Progressive Depression (*n* = 764)		Trajectory of Consistent Depression (*n* = 182)		
	M	SD	M	SD	M	SD	F/*p*
Depressive symptoms	2.66	3.23	7.02	4.99	15.18	6.61	
Protective factors							
Self-health perception	3.65	1.04	3.02	1.03	2.29	0.95	193.88 **
Exercise behavior	0.51	0.50	0.40	0.49	0.33	0.47	19.47
Family living satisfaction	4.19	0.71	3.93	0.78	3.56	0.82	72.32 **
Cognitive function _Recall	4.86	2.19	4.56	2.31	3.55	2.19	28.25 **
Social participation	0.68	0.92	0.50	0.80	0.34	0.72	18.40 **
Financial satisfaction	3.36	0.82	3.03	0.84	2.39	0.88	127.37
Risk factors							
Number of chronic diseases	0.78	1.03	1.24	1.31	1.92	1.61	94.18
Pain	1.29	0.59	1.60	0.79	2.28	1.07	172.77
Substance use behavior	0.69	0.90	0.59	0.87	0.46	0.76	6.87
IADL difficulty	0.24	1.13	0.72	2.00	2.45	4.31	114.74

Note. The 1996 Taiwan Longitudinal Survey on Aging (TLSA)—a total of 2344 ≥ 50-year-old population-representative citizens from three samplings completed the survey. ** p < 0.01.

**Table 4:** Differences in protective and risk factors for depression trajectory grouping among varied cognitive function groups.

Grouping by the Ability to Correctly Recite from Memory	Normal Cognition									Mild Cognitive Impairment (MCI)								
	Trajectory of Maintained Mood		Trajectory of Progressive Depression		Trajectory of Consistent Depression					Trajectory of Maintained Mood		Trajectory of Progressive Depression		Trajectory of Consistent Depression				
104 Years of Variables	G1		G2		G3		Total			G1		G2		G3		Total		
	M	SD	M	SD	M	SD	M	SD	F	M	SD	M	SD	M	SD	M	SD	F
Protective factors																		
Self-health perception	3.83	0.97	3.25	1.02	2.42	1.06	3.65	1.04	44.97 ***	3.50	1.09	2.99	1.00	2.34	0.94	3.18	1.10	18.55 ***
Exercise behavior	0.51	0.50	0.40	0.49	0.23	0.43	0.48	0.50	7.07 **	0.55	0.50	0.37	0.49	0.24	0.44	0.45	0.50	7.38 **
Family living satisfaction	4.23	0.70	3.98	0.78	3.44	0.71	4.15	0.74	20.70 ***	4.17	0.78	3.94	0.79	3.72	0.75	4.03	0.79	5.58 **
Social participation	0.73	0.92	0.49	0.83	0.38	0.80	0.66	0.90	6.32 **	0.58	0.82	0.52	0.77	0.24	0.44	0.52	0.78	2.27
Financial satisfaction	3.40	0.84	3.07	0.85	2.31	0.84	3.29	0.87	28.93 ***	3.36	0.71	3.14	0.75	2.41	0.91	3.18	0.79	19.79 ***
Risk factors																		
Number of chronic diseases	0.61	0.91	0.94	1.03	1.58	1.53	0.72	0.98	18.63 ***	0.73	1.01	1.28	1.35	1.71	1.56	1.05	1.26	11.79 ***
Pain	1.24	0.52	1.52	0.79	2.31	1.09	1.34	0.65	47.07 ***	1.35	0.62	1.64	0.72	2.55	1.15	1.58	0.80	34.24 ***
Substance use behavior	0.67	0.89	0.58	0.86	0.58	0.90	0.64	0.88	0.66	0.40	0.72	0.27	0.62	0.28	0.65	0.34	0.67	1.32
IADL difficulty	0.07	0.33	0.24	0.91	0.88	1.58	0.13	0.61	27.40 ***	0.29	0.95	0.61	1.16	2.04	3.66	0.58	1.55	16.58 ***

** *p* < 0.01, *** *p* < 0.001.

## Data Availability

The data that support the findings of this study are available from the Health Data Science Center, Taiwan, but restrictions apply to the availability of these data, which were used under license for the current study and so are not publicly available. Data are, however, available from the corresponding author upon reasonable request and with permission of the Taiwan Ministry of Health and Welfare.

## Supplementary Materials

The following supporting information can be downloaded at https://www.mdpi.com/article/10.3390/healthcare12242568/s1, Supplementary Figure S1: Flow diagram of the study participants; Supplementary Table S1: Covariate predicting depression trajectory memberships and protect and risk factor using Multinomial Logistic Regression.
