# Supplementary material for: Long-Term Determinants of Depression Mood: A 19-Year Follow Up of 2344 Middle-Aged and Older Adults
Source: Healthcare (Basel). 2024 Dec 20;12(24):2568. doi: 10.3390/healthcare12242568 (PMC11675579; doi:10.3390/healthcare12242568)
Supplement: Supplementary file 1 [file healthcare-12-02568-s001.zip › healthcare-3353250-supplementary.pdf]

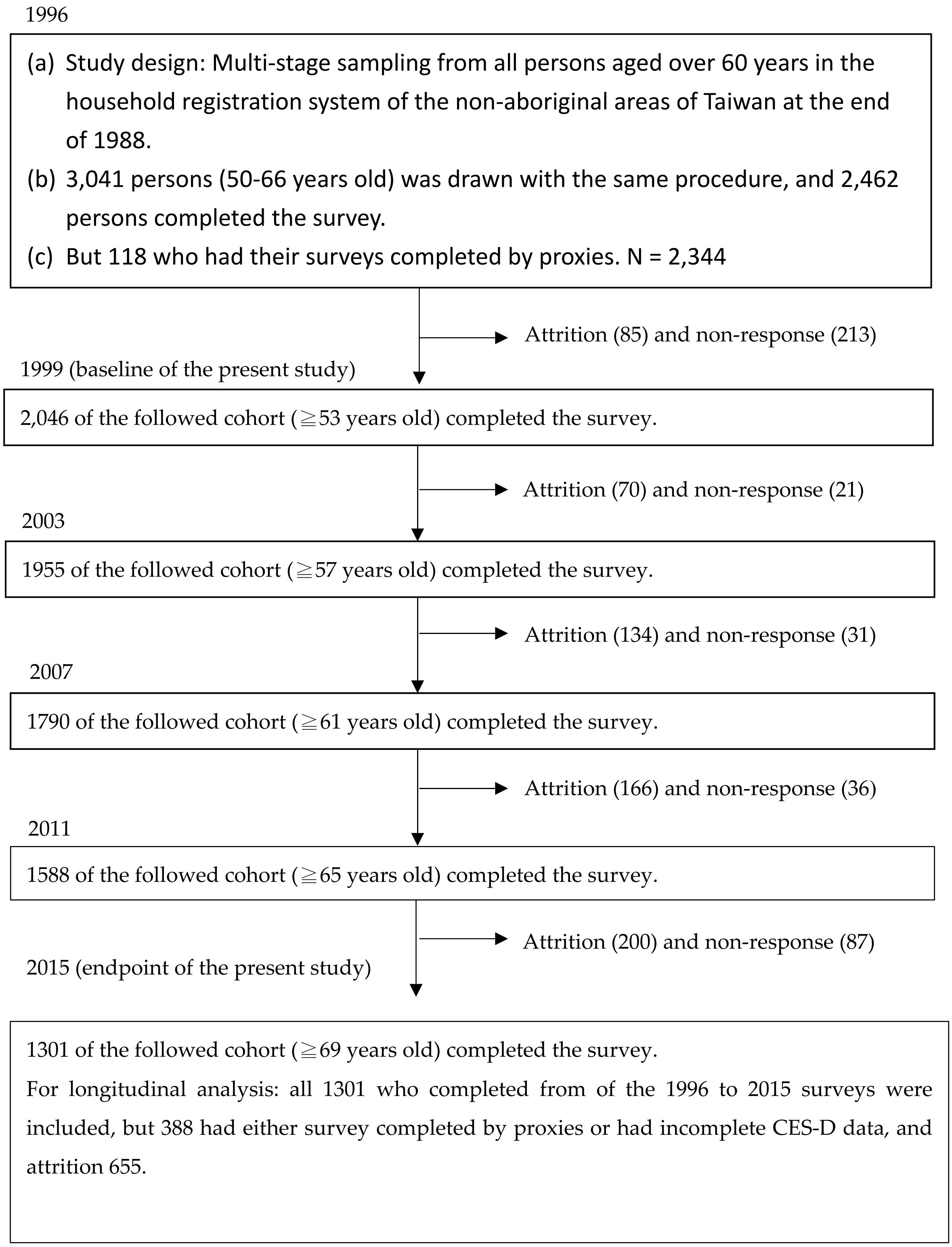

Supplementary Figure S1. Flow diagram of study participants

Supplementary Table S1. Covariate predicting depression trajectory memberships and protect and risk factor using Multinomial Logistic Regression.

	Trajectory 2 vs. Trajectory 1 (G2 VS G1)							Trajectory 3 vs. Trajectory 1 (G3 VS G1)						
Variables	β	SE	Wald	*P*	OR	95 % C.I		β	SE	Wald	*P*	OR	95 % C.I	
						L	U						L	U
Intercept	2.40	0.42	32.57	.000				5.06	0.76	44.08	.000			
Time constant variables														
SEX (female)	-0.54	0.13	17.75	.000	0.58	0.45	0.75	-0.75	0.26	8.47	.004	0.47	0.28	0.78
Education (literacy)	0.41	0.12	11.34	.001	1.51	1.19	1.92	0.50	0.22	4.94	.026	1.64	1.06	2.55
Live alone	-0.47	0.22	4.33	.037	0.63	0.40	0.97	-0.25	0.38	0.43	.510	0.78	0.37	1.64
Time varying variables														
Protective factors														
Self-health perception	-0.30	0.06	28.12	.000	0.74	0.66	0.83	-0.63	0.13	24.97	.000	0.53	0.41	0.68
Exercise behavior	-0.29	0.10	7.82	.005	0.75	0.61	0.92	-0.48	0.21	5.36	.021	0.62	0.41	0.93
Family living satisfaction	-0.27	0.07	14.72	.000	0.76	0.66	0.87	-0.77	0.13	34.24	.000	0.46	0.36	0.60
Cognitive function _Recall	0.02	0.02	0.64	.425	1.02	0.97	1.06	-0.14	0.05	7.98	.005	0.87	0.79	0.96
Social participation	-0.08	0.06	1.86	.173	0.92	0.82	1.04	-0.18	0.14	1.47	.225	0.84	0.63	1.11
Financial satisfaction	-0.30	0.06	22.80	.000	0.74	0.65	0.84	-0.98	0.12	63.10	.000	0.38	0.30	0.48
Risk factors														
Number of chronic disease	0.17	0.05	13.29	.000	1.19	1.08	1.30	0.34	0.07	21.62	.000	1.41	1.22	1.62
Pain	0.30	0.08	14.62	.000	1.35	1.16	1.58	0.67	0.12	31.10	.000	1.95	1.54	2.46
Substance use behavior	0.14	0.07	4.40	.036	1.16	1.01	1.32	0.14	0.14	0.96	.327	1.15	0.87	1.53
IADL difficulty	0.07	0.04	4.18	.041	1.08	1.00	1.16	0.13	0.04	9.00	.003	1.14	1.05	1.24

Note. G1 = Trajectory of maintained mood, G2 = Trajectory of progressive depression, G3 = Trajectory of consistent depression, OR (95%CI) = odds ratio (95%confidence interval).
